# Host size matters for reproduction: Evolution of spawning preference and female reproductive phenotypes in mussel‐symbiotic freshwater bitterling fishes

**DOI:** 10.1002/ece3.11142

**Published:** 2024-03-11

**Authors:** Hee‐kyu Choi, Hyuk Je Lee

**Affiliations:** ^1^ Molecular Ecology and Evolution Laboratory, Department of Biological Science, College of Science and Engineering Sangji University Wonju Korea

**Keywords:** coevolutionary arms race, coextinction, host preference, morphological adaptation, mussel‐symbiotic bitterling, two‐sided host‐parasitism

## Abstract

Bitterling fishes evolve an idiosyncratic symbiosis with freshwater mussels, in which they are obligated to spawn in the gills of mussels for reproduction. In recent years, freshwater mussel populations have been drastically diminishing, due to accelerating anthropogenic impacts, which can be large threats to the risk of bitterling's extinction cascade (i.e. ‘coextinction’). The host mussel size may be an important factor driving the adaptation and evolution of bitterling's reproductive phenotypes. Here we examined the host size preference and morphological adaptation of female bitterling to the host size from 17 localities at the Han River in Korea. Using our developed molecular‐based species identification for bitterling's eggs/larvae inside the mussels, we further determined the spawning patterns of seven bitterling species. Mean length of spawned mussels (*N* = 453) was significantly larger than that of unspawned mussels (*N* = 1814), suggesting that bitterling prefers to use larger hosts as a spawning ground. Spawning probability was clearly greater as mussel size increases. Results of our reciprocal transplant experiments do provide some evidence supporting the ‘bitterling's larger host preference’ hypothesis. Interspecific competition appeared to be intense as two fish species often spawned eggs in the same mussel individuals simultaneously. Longer ovipositor and more elongated egg may evolve in females of *Tanakia signifer* in response to larger host environments. The observed bitterling's spawning preference for large‐sized mussels may evolve perhaps because of the fitness advantage in relation to the offspring survival. Our findings further inform on the development of effective conservation and management strategy for the endangered bitterling fishes.

## Graphical abstract

Bitterling fishes evolve an idiosyncratic symbiosis with freshwater mussels, in which they are obligated to spawn in the gills of mussels for reproduction. The observed bitterling's spawning preference for large‐sized mussels may evolve perhaps because of the fitness advantage in relation to the offspring survival. Different reproductive strategies (or fecundity) evolve in six Korean bitterling fishes. Our findings further inform on the development of effective conservation and management strategy for the endangered bitterling fishes.

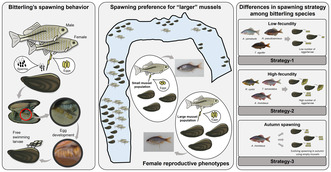

## 1 INTRODUCTION

Symbiosis involves any kind of interactions between members of two different species, such as mutualism, commensalism and parasitism (Thompson, 2009). Mutualism occurs when two interacting counterparts benefit from each other, commensalism arises when one species benefits and the other unaffected, and parasitism unilaterally exploits one species (Paracer & Ahmadjian, 2000). In particular, brood parasitism, the exploitation by the brood parasite of the parental care of the host, is rare but taxonomically widespread, being found in birds (Abolins‐Abols & Hauber, 2018), fishes (Aldridge, 1999) and insects (Thompson & Pellmyr, 1991). While parasites tend to adapt to the hosts, the hosts typically counter‐adapt alleviating fitness loss burdened by parasites. These reciprocal interactions could lead to a co‐evolutionary arms race between the parasites and the hosts (Davies et al., 1989).

**FIGURE 1:**
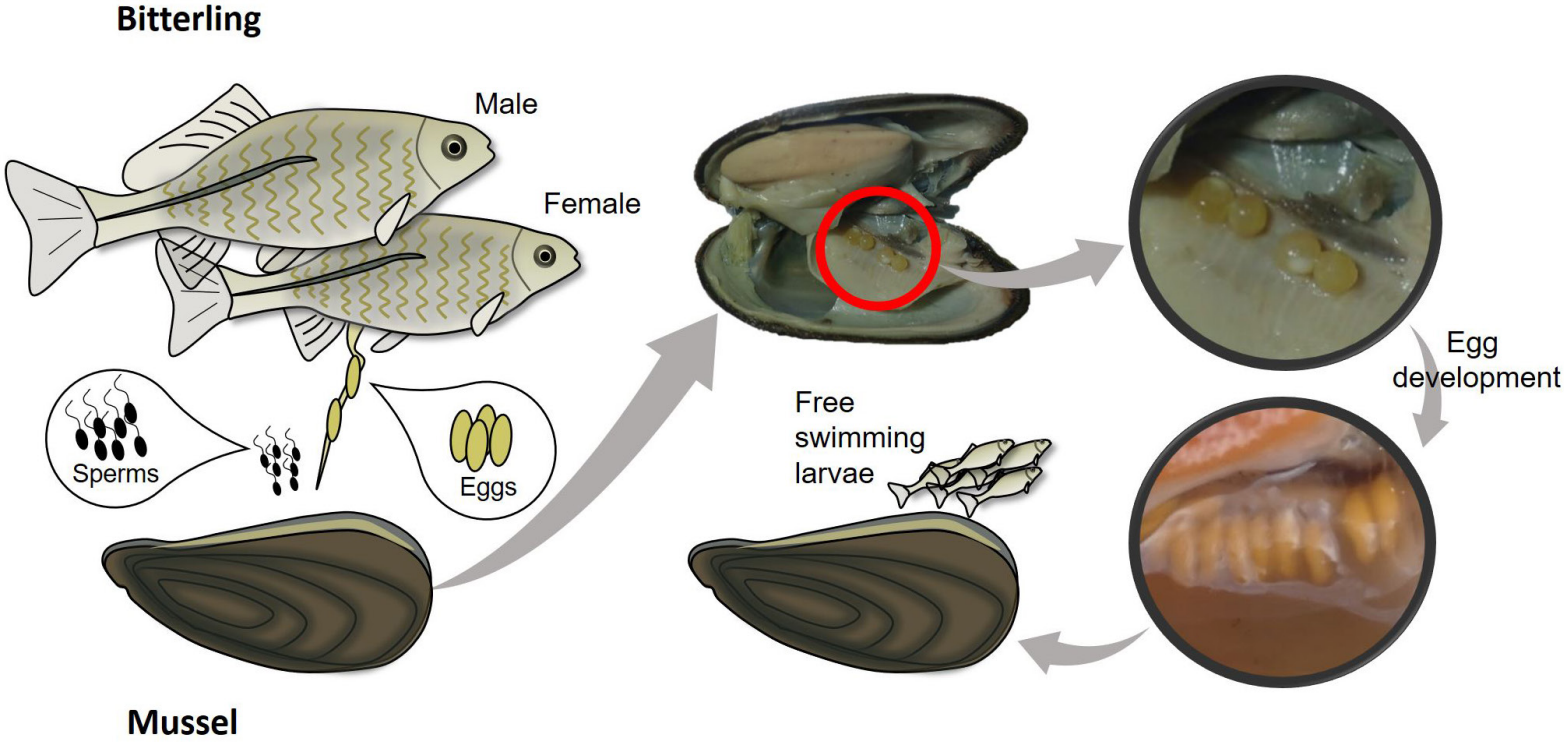
A diagram illustrating the symbiotic relationship between freshwater bitterling fishes and mussels. Bitterlings are obligated to spawn in the gills of the mussels for reproduction. The fertilized eggs develop inside the mussels and eventually leave the hosts for approximately a month as free swimming larvae.

Brood parasitism has been studied regarding host defence, reproductive behaviour of parasites and species‐specific host preference in some groups of animals, such as birds (Abolins‐Abols & Hauber, 2018), insects (Abolins‐Abols & Hauber, 2018; Faeth, 1991; Thompson & Pellmyr, 1991) and also freshwater fishes (Kim et al., 2004). Faeth (1991) observed a preference of moth *Cameraria* sp. (Lepidoptera: Gracillariidae) for a larger host leaf, which might indicate that a relatively larger leaf as a habitat could offer enhanced fitness benefits for larval development. In freshwater fishes, bitterling species (Cyprinidae, Acheilognathinae) evolved a symbiosis with mussels, in which they are obligated to spawn in the gills of mussels (Unionidae) (Figure 1) (Aldridge, 1999). Whilst male bitterlings defend a territory around mussels for attracting females, female bitterlings use their extended ovipositors to lay the eggs onto the demibranchs (gills) of mussels through the mussel's exhalant siphon. The eggs are fertilized by male's ejaculated sperms through the mussel's inhalant siphon. The fertilized eggs then develop inside the mussels for nearly a month, eventually leaving the hosts as actively swimming larvae (Figure 1) (Smith et al., 2004). Fitness benefits for the bitterling fishes would be apparent as the use of mussels as a spawning/nursery ground ensures high offspring survival and no need for parental care (Aldridge, 1999).

Yet, whether one or both sides benefit from the interaction between bitterlings and host mussels remains unclear (Mills & Reynolds, 2003; Paracer & Ahmadjian, 2000). In previous studies, the relationship between bitterlings and mussels has generally been considered as mutualistic since the fishes use mussels as a spawning/nursery substrate, and the mussels (family Unionidae) use bitterlings (and also other fishes) as a vehicle for the dispersal of their larvae called glochidia (Dillon, 2000; Wheeler, 1978). Hence, both sides could profit from each other. However, more recent studies found little evidence for the mutualistic relationship and suggested an alternative hypothesis that bitterling spawning may have negative effects on mussels. Bitterling's spawned eggs/larvae can impair the respiration and reproduction rates of mussels (Mills et al., 2005; Mills & Reynolds, 2002; Reichard et al., 2010). Glochidia infection rate was even higher in other fishes such as goby and cyprinid than in bitterlings (Reichard et al., 2010). These observations argue against the hypothesis of mutualistic relationship between bitterlings and mussels. The bitterling‐mussel association was suggested as asymmetric, that is, a ‘host–parasite’ relationship (Brian, 2022; Reichard, Przybylski, et al., 2007). More recently, the relationship between bitterling fishes and unionid mussels was suggested as a ‘two‐sided host–parasite association’ in which both parasitize reciprocally (Methling et al., 2018; Rouchet et al., 2017). Still, the ultimate evolutionary origins and ecological explanations as to the symbiotic interaction between bitterlings and mussels as well as proximate mechanisms of bitterling's host preference and female phenotypic response as a function of the host size remain poorly understood.

Freshwater mussel populations have sharply been decreasing worldwide, perhaps due to increasing anthropogenic disturbances, such as habitat destruction and fragmentation, for example, construction of hydropower dams and weirs (Nakamura et al., 2023). This collapse of mussel populations might result in increasing interspecific as well as intraspecific resource competition among bitterling fishes for spawning grounds, which may in turn exacerbate bitterling populations (Hata et al., 2021; Kanoh, 2000; Onikura et al., 2006). Massive decline of these hosts could trigger the extirpation of bitterling species (i.e. ‘coextinction’) (Colwell et al., 2012; Modesto et al., 2018). Moreover, a decrease in breeding substrate may cause hybridization between different species using the same breeding substrate (Hata et al., 2021; Hubbs, 1955). In Northeast Asia, including Japan and Korea, two to six or seven different bitterling species usually coexist in streams/rivers, and different species sometimes spawn in the same host mussel individuals simultaneously, probably due to high levels of resource competition (Choi & Lee, 2019; Kim et al., 2016; Kitamura, 2007; Seo et al., 2023). Spawning patterns of four bitterling species (*Acheilognathus rhombeus*, *Acheilognathus tabira tabira*, *Tanakia lanceolate* and *Tanakia limbata*) inhabiting Japanese rivers have been studied based on RFLP (restriction fragment length polymorphism)‐based species identification for eggs/larvae inside the mussels (Hata et al., 2019; Kitamura, 2007; Uemura et al., 2018). However, the spawning patterns of Korean bitterling species have been investigated solely based on morphology‐based species identification of eggs/larvae, although 15 species belonging to three genera have been reported up to now (Kim et al., 2014, 2018; Park et al., 2018). This may potentially bring artefacts given a difficulty in morphological species identification of bitterling's egg/larvae as egg shape may change according to life stages and environmental conditions (Kitamura, 2007; Kitamura et al., 2012). Our previous study developed a RFLP marker for discriminating among three Korean bitterling species (*Tanakia signifer*, *Acheilognathus yamatsutae* and *Rhodeus uyekii*) (Choi & Lee, 2018). By extending our previous RFLP into distinguishing eight bitterling species, we here aimed to understand the spawning frequencies, preferences and reproductive ecology of those fishes at the species level, particularly in association with the host body size.

In the present study, we investigated the spawning patterns of seven bitterling fish species (*A. rhombeus*, *A. yamatsutae*, *Rhodeus ocellatus ocellatus*, *Rhodeus pseudosericeus*, *R. uyekii*, *T. lanceolatus* and *T. signifer*) with respect to the host size from 17 localities in the Han River basin in Korea. The specific objectives of this study were as follows: (1) to examine spawning frequencies and preferences of these bitterling fishes as a function of the mussel (host)'s shell length (i.e. body size) at the community as well as the species levels; (2) to test for the observed hypothesis that the bitterling fishes prefer to use larger mussels for spawning by using larger hosts unfamiliar to the fishes (non‐native from a foreign place) through reciprocal transplant experiments; (3) to examine whether the spawning positions of inner or outer gills of each bitterling species are species specific and (4) to examine the morphological adaptation of female's reproductive traits in *T. signifer*, such as ovipositor length and egg shape, in response to the host size. We hypothesized that ‘bitterling's larger host preference’ and ‘female's longer ovipositor and more elongated egg in larger hosts’ may evolve as those traits are selected for in response to the host environments. The findings of our study will provide an insight into the evolutionary trajectory and process in an unusual symbiosis and the reproductive ecology of the Korean bitterling fishes in general. The results of this study also inform on conservation implications for endangered bitterling fish species, particularly *T. signifer* and *R. pseudosericeus*, which have been designated as endangered wild species class II for legal protection by the Ministry of Environment of Korea.

## 2 MATERIALS AND METHODS

### 2.1 Study sites and sampling

**FIGURE 2:**
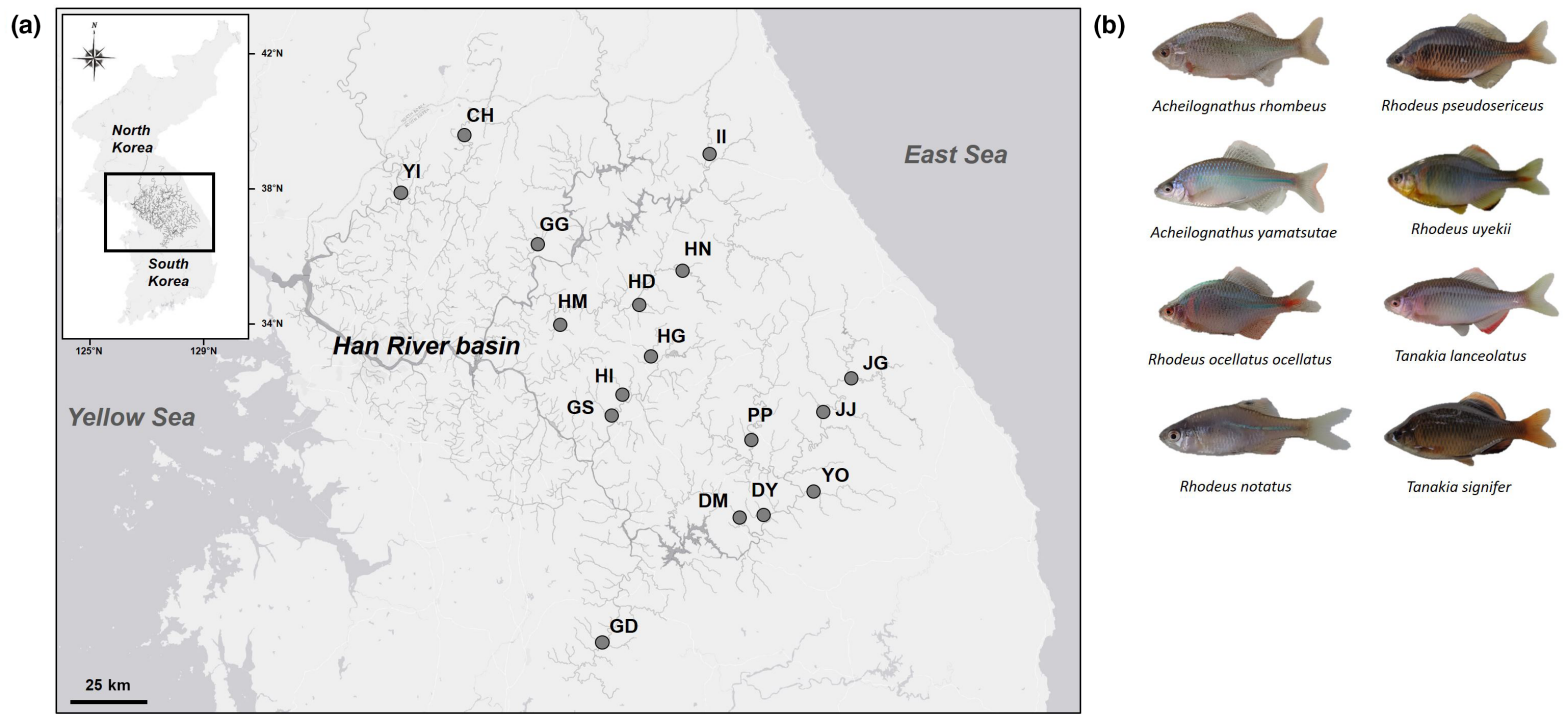
Sampling localities of Acheilognathinae bitterling fishes and freshwater mussels from 17 sites in the Han River basins from South Korea. (a) The study sites included Imjin‐Hantan River (YI, CH), North Han River (II, GG, HN, HD, HM) and South Han River (HG, HI, GS, JG, JJ, PP, YO, DY, DM, GD). (b) Eight Acheilognathinae bitterling fish species (*A. rhombeus*, *A. yamatsutae*, *R. notatus*, *R. ocellatus ocellatus*, *R. pseudosericeus*, *R. uyekii*, *T. lanceolatus* and *T. signifer*) observed. Detailed information on each locality and location codes are given in Table S1.

The field survey was conducted at 17 localities from the Han River basins (including the Imjin‐Hantan River, the North Han River and the South Han River) in South Korea between April and May of 2019 (Figure 2a, Table S1). Bitterling fish individuals were collected using both kick net (mesh size, 4 × 4 mm) and cast net (7 × 7 mm). At each site, the coexisting mussels were collected by hand and also with a kick net. Mussel density was estimated by performing a random sampling using seven replicates of a 1 × 1 m quadrat in a 2 m interval along a transect line. We measured the shell length, shell height and shell width to the nearest 0.01 mm by using a digimatic calliper (0–150 mm; Mitutoyo, Japan) on the collected mussels to test for the bitterling's host size preference. The presence/absence of bitterling's eggs/larvae inside the mussels was checked by opening a 1 cm gap between the shells of the mussels using a mussel‐opening device and forcep (Kitamura, 2005). Only the mussels spawned by bitterlings were stored in 50 mL falcon tubes containing 99% ethanol for subsequent genetic analyses (for species identification of eggs/larvae). The unspawned mussels were released back to the streams where they were caught immediately after the measurements. In the field surveys, a total of eight bitterling species (*A. rhombeus*, *A. yamatsutae*, *Rhodeus notatus*, *R. ocellatus ocellatus*, *R. pseudosericeus*, *R. uyekii*, *T. lanceolatus* and *T. signifer*) were identified (Figure 2b). All the species except *A. rhombeus* (an autumn spawner) are spring spawners (Kim, 1997; Kim & Park, 2002). Four species of mussels were identified (*Nodularia breviconcha*, *Nodularia douglasiae*, *Lanceolaria acrorrhyncha* and *Anodonta arcaeformis flavotincta*) (Lopes‐Lima et al., 2020). Of these, *N. breviconcha* (*N* = 2188; 95.76%) was predominant and *N. douglasiae* (*N* = 79; 3.46%) was subdominant, regardless of sampling sites. *Lanceolaria acrorrhyncha* (*N* = 3; 0.13%) and *A. arcaeformis flavotincta* (*N* = 15; 0.65%) were rare and thus excluded from subsequent analyses. The two species of the genus *Nodularia* are morphologically similar but with some distinctions in morphological characteristics, such as shell size, thickness and inner surface colour (Kwon, 1990). *Nodularia douglasiae* usually exhibits a larger and thicker shell relative to *N. breviconcha*, with some protrusions/spines present on the upper part of the outer shell and more whitish coloured inner shell surface (*N. breviconcha*: more yellowish coloured) (Kwon, 1990; Hee‐kyu Choi, personal observation). Field work was conducted under collection permit Nos.: 2019‐07, 2019‐11, 2020‐09 and 2020‐20 granted by the Regional Environmental Offices of the South Korean government.

### 2.2 Reciprocal transplant experiments

**FIGURE 3:**
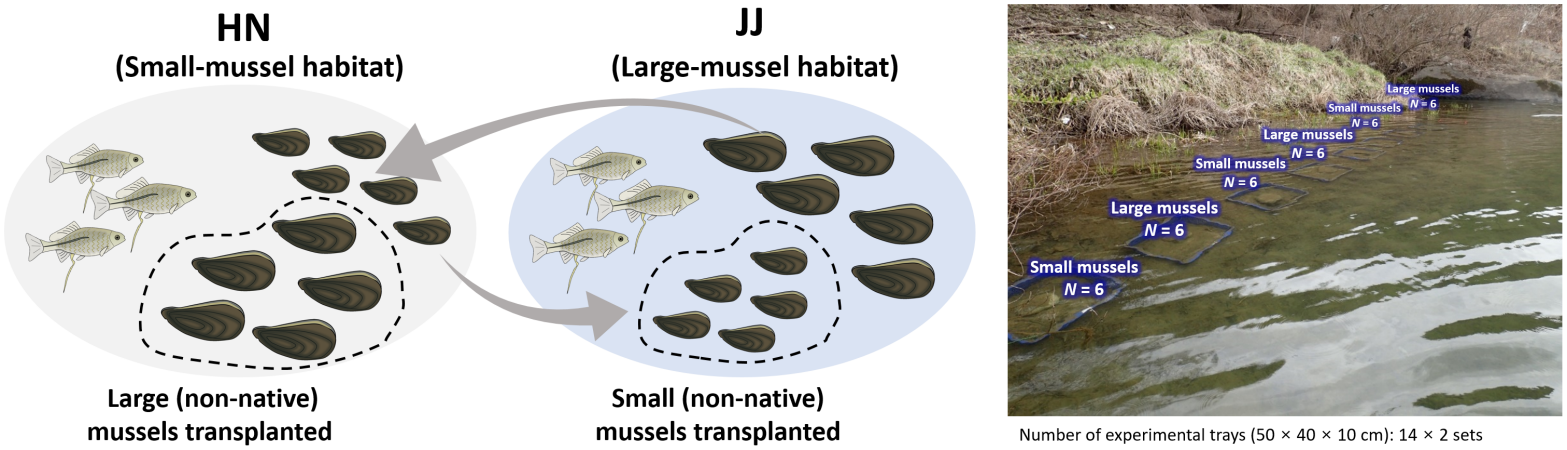
Graphical representation illustrating the reciprocal transplant experiments conducted for between HN (a small‐mussel habitat) and JJ (a large‐mussel habitat) to test for the hypothesis of the bitterling's preferences for larger hosts as a spawning ground.

Reciprocal transplant experiments were performed to test the observed hypothesis that the bitterling fishes prefer larger mussels for spawning. We reciprocally transplanted small‐ and large‐sized mussels (*N. breviconcha*) from two populations (small mussel population: HN, mean shell length = 29.71 ± 4.89 [standard deviation; SD] mm; large mussel population: JJ, mean shell length = 42.51 ± 6.43 mm) to determine if bitterlings maintain the large‐host preference for spawning in larger (but unfamiliar) mussels from a foreign place or spawning in familiar (but smaller) hosts at home (Figure 3). We collected mussels that had not been spawned by bitterling fishes during February–March before the spawning season (April–June) (Baek & Song, 2005; Uchida, 1939). In order to completely exclude the possibility that the mussels had eggs/larvae, they were transported to the laboratory and accommodated in the experimental tanks (45 × 30 × 30 cm) for a month to make sure all eggs/larvae were ejected (if present). For the field experiments, plastic tray containers with mesh (50 × 40 × 10 cm), which allowed a water flow, were anchored at each site to hold sands/sediments with the experimental mussels, which mimicked the same microhabitat environments across the experimental plots (Kim et al., 2013). Fourteen plastic boxes (accessible with bitterling fishes) were placed at an interval of 2 m in each experimental site (HN, JJ), and one more batch of the same 14 plots was placed at another point within both HN and JJ localities. Six to seven individuals of small and large mussels per plot were placed in the plastic box and collected after 1 month of experimental periods in April–May of 2019, 2020 and 2021 years. All the experimental mussels were transported to the laboratory and checked for the presence/absence of eggs/larvae and number of eggs/larvae was counted if present. The eggs/larvae found in the reciprocal translocation experiments were not genetically analysed for species identification, although only one species (*T. signifer*) and two species (*T. signifer* and *A. yamatsutae*) were observed in HN and JJ, respectively, based on the results of RFLP for eggs/larvae in each site.

### 2.3 Species identification of bitterling's eggs/larvae

Bitterling's eggs/larvae inside the gills of host mussels were secured for the molecular‐based species identification. Prior to extracting eggs/larvae, their positions inside the mussels were checked and recorded by classifying the slots into four gill spaces (two [left/right] inner and two outer spots). Eggs were further grouped by their shape (e.g. fusiform, bulb like, pear, ovoid) following a previous study (Park et al., 2018). Differences in egg shape may represent different species; however, it may change with developmental stage (Kim, 2020). They were then stored in 1.5 mL micro‐centrifuge E‐tube containing 99% ethanol for a given host mussel. In each mussel, eggs/larvae with the same shape (indicating the same developmental phase) located at the same gill positions were presumed to be a clutch from the same parental fishes (Hata et al., 2021). Three eggs, larvae, or fry randomly chosen from each of the clutches were genetically analysed (total number of samples analysed [*N* = 514]). Genomic DNA was extracted from the eggs and larva tissues (~2 mm) using a G‐spin Total DNA Extraction Kit (iNtRON Biotechnology, Korea). Mitochondrial DNA (mtDNA) cyt *b* (cytochrome *b*) region (1105 bp) was amplified by PCR with the forward primer cyt *b*‐F, 5′‐GAYTTGAAGAACCATCGTTGT‐3′, and the reverse primer cyt *b*‐R, 5′‐CTTCGGATTACAAGACCGATG‐3′ (Chang et al., 2014). The reaction volume of 15 μL was comprised of 10 × Green buffer (Thermo Fisher Scientific, Waltham, MA, United States), 20 mM of each dNTP (Bio Basic Inc., Canada), 0.6 mM of each of the forward and reverse primers, 0.2 U of Taq polymerase (Thermo Fisher Scientific, United States) and 0.85–57.24 ng/μL of DNA template. The following thermal conditions were applied: initial denaturation at 94°C for 4 min followed by 35 cycles of denaturation at 94°C for 1 min, annealing at 55°C for 1 min and extension at 72°C for 1 min, followed by a final extension at 72°C for 5 min. The PCR products were genotyped by RFLP using two enzymes *Hin*P1I (G'CGC) and *Rsa*I (GT'AC) (Enzynomics, Korea). We developed RFLP markers by using information on DNA sequences deposited in GenBank. To consider intraspecific variation for each species, we used DNA sequences from five adult individuals per species collected in the field. The overall scheme behind the RFLP‐based species identification was summarized in Figure S1 and Table 1. Briefly, *Hin*P1I (G'CGC) produced two, three, four and five fragments of different sizes differentiating five bitterling species (*R. notatus*, *R. pseudosericeus*, *R. uyekii*, *T. signifer* and *T. lanceolatus*) except *A. rhombeus*, *A. yamatsutae* and *R. ocellatus ocellatus*. *Rsa*I (GT'AC) further discriminated the remaining three bitterling fishes (Figure S1, Table 1). We incubated 5.0 μL of the PCR products with 2 U of restriction enzyme (10 U/μL), 1 μL of 1 × Ezbuffer and 10 μL of sterilized water at 37°C overnight. The reaction products were electrophoresed with a 100 bp DNA Ladder (iNtRON Biotechnology, Korea) for 20 min at 100 V on 2% agarose gels stained with RedSafe (iNtRON Biotechnology). We then determined the species for the analysed samples based on the resulting fragment patterns. To verify the accuracy of our developed RFLP markers, randomly selected 31 specimens [GD (*N* = 8), DM (*N* = 4), YI (*N* = 19)] were sequenced for mtDNA cyt *b* (776–1013 bp) and found to be 100% matched with the RFLP species identification (data not shown).

**TABLE 1:** DNA fragment sizes expected from two restriction enzymes (*Hin*P1l; G'CGC, *Rsa*l; GT'AC) treatments after mtDNA cyt *b* (1105 bp) PCR reaction for eight different species of the Acheilognathinae bitterling fishes.

	PCR product sizes (bp)	Restriction enzyme	Species	Expected fragment lengths (bp) after digestion with respective restriction enzymes				
1st	Cyt *b* 1105	*Hin*P1I (G'CGC)	*A. rhombeus*	241	864			
			*A. yamatsute*	241	864			
			*R. ocellatus ocellatus*	241	864			
			*R. notatus*	241	679	185		
			*R. pseudosericeus*	241	839	25		
			*R. uyekii*	241	168	696		
			*T. lanceolatus*	241	218	252	369	25
			*T. signifer*	241	821	18	25	
2nd	Cyt *b* 1105	*Rsa*I (GT'AC)	*A. rhombeus*	378	88	417	168	54
			*A. yamatsute*	337	546	168	54	
			*R. ocellatus ocellatus*	337	105	441	222	
			*R. pseudosericeus*	291	391	201	222	
			*T. signifer*	883	222			

### 2.4 Female spawning phenotypes in relation to the mussel size

The reproductive traits (e.g. ovipositor length, egg shape) of female *T. signifer* were measured and analysed at each site to test for the morphological adaptation incurring in response to the host shell size. This particular species was chosen for this experiment as it was the most widely distributed and observed at 15 of 17 sites (except HG, HI) and also it is an endangered species. Standard body length (SL) and ovipositor length (OL) were measured to the nearest 0.1 mm for 1–30 female individuals per population (mean number = 12.13 ± 6.70). By gently squeezing sexually matured female's abdomen, mature eggs were released through the ovipositor. The collected bitterlings were released back to the sites where they were caught immediately after measurements and egg extraction. The ovipositor ratio was used for the formal analysis by calculating OL/SL values (length of ovipositor relative to standard body length) to correct for the differences according to the age (size) of the female individuals. Egg length (EL) and egg diameter (ED) of the extracted eggs were measured using a stereoscopic microscope (Olympus‐SZ61, Japan) for 2–6 female individuals per population (mean number = 4.86 ± 0.95), and EL/ED values were calculated to determine egg shape (Kitamura et al., 2012).

### 2.5 Data analyses

Statistical analyses were conducted using parametric or non‐parametric methods in SPSS version 25.0 (SPSS Inc., United States), as appropriate after testing for the normality using Shapiro–Wilk test. Differences in shell length (i.e. body size) between spawned and unspawned mussel groups were assessed using non‐parametric Mann–Whitney *U* tests for the entire pooled mussel community as well as for each locality. The size of all collected mussels was divided into 5 mm intervals to investigate if proportion (%) of spawned mussels increased as increasing size classes. We then created a binary variable, categorizing each mussel gill inside of spawned or unspawned of eggs/larvae (spawned = 1 or unspawned = 0), and simple logistic regression analysis was conducted to determine the relationship between the predictor (shell length of mussels) and a predicted variable, probability of spawning by calculating the odds ratio with a 95% confidence interval (CI). For the reciprocal transplant experiments, Student's independent *t*‐tests were performed to compare mean spawning frequency and mean number of spawned eggs/larvae between two groups comprising the large mussel habitat (JJ) and the small mussel habitat (HN). The chi‐square (*χ* ^2^) tests were used to determine whether the gill positions (inner or outer gills) of spawned eggs/larvae were non‐random in seven bitterling species. To examine the effects of the host size on bitterling's fecundity and female's reproductive phenotypes, three different linear regression analyses were performed to analyse the relationships between the mussel shell length and the number of eggs/larvae, OL/SL (ovipositor ratio) and EL/ED (egg shape ratio). The relationships between proportion of spawning (% of spawned mussels), and mussel density and number of coexisting bitterling species were also analysed using Pearson's correlation analyses. Furthermore, two non‐parametric Kruskal–Wallis tests were performed to determine whether there were differences in mean number of eggs/larvae per mussel and also in mean shell length of spawned mussels among the six bitterling species. Dunn's multiple comparison tests were carried out as post hoc tests and the significance level was adjusted using a Bonferroni correction (*p* < .05).

## 3 RESULTS

### 3.1 Spawning frequencies and preferences as a function of the host mussel size

**FIGURE 4:**
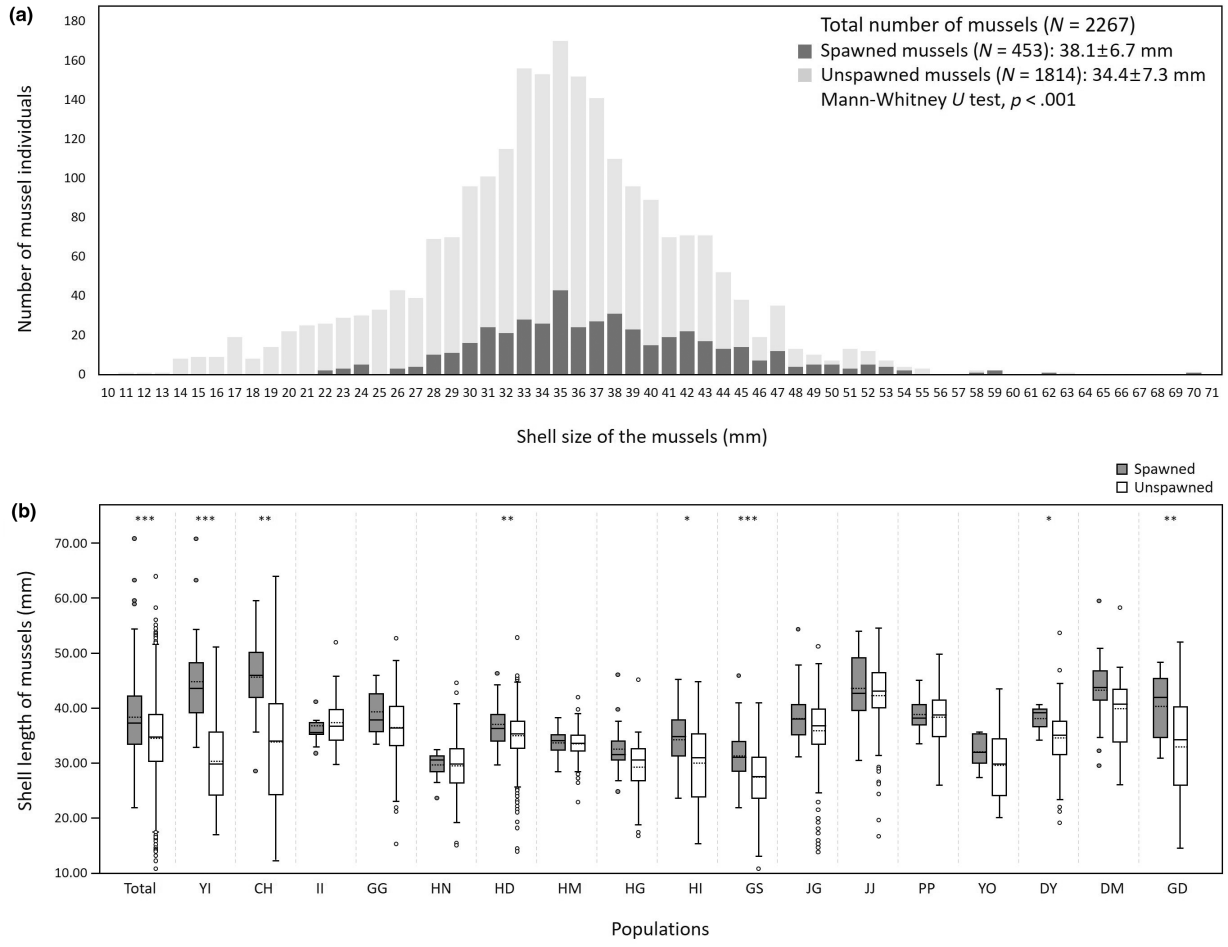
Size‐frequency distribution of two Unionidae (*Nodularia douglasiae*, *Nodularia breviconcha*) at 17 study sites (*N* = 2267). (a) Vertical bars in light grey represent unspawned mussels (having no bitterling's eggs/larvae) (*N* = 1814; size mean = 34.43, ± SD = 7.25 mm) and those in dark grey represent spawned mussels (having bitterling's eggs/larvae) (*N* = 453; 38.07 ± 6.73 mm). (b) Box plot illustrating differences in shell length between mussels with eggs/larvae (spawned; grey box) and without eggs/larvae (unspawned; white box) at the 17 study sites. The line within the box is the median; the dotted line within the box is the mean; the box marks the 25th and 75th percentiles; the whiskers mark the 10th and 90th percentiles and the circles represent outliers. Statistical analyses were performed with non‐parametric Mann–Whitney *U* tests. *p < .05, **p < .01, ***p < .001.

**FIGURE 5:**
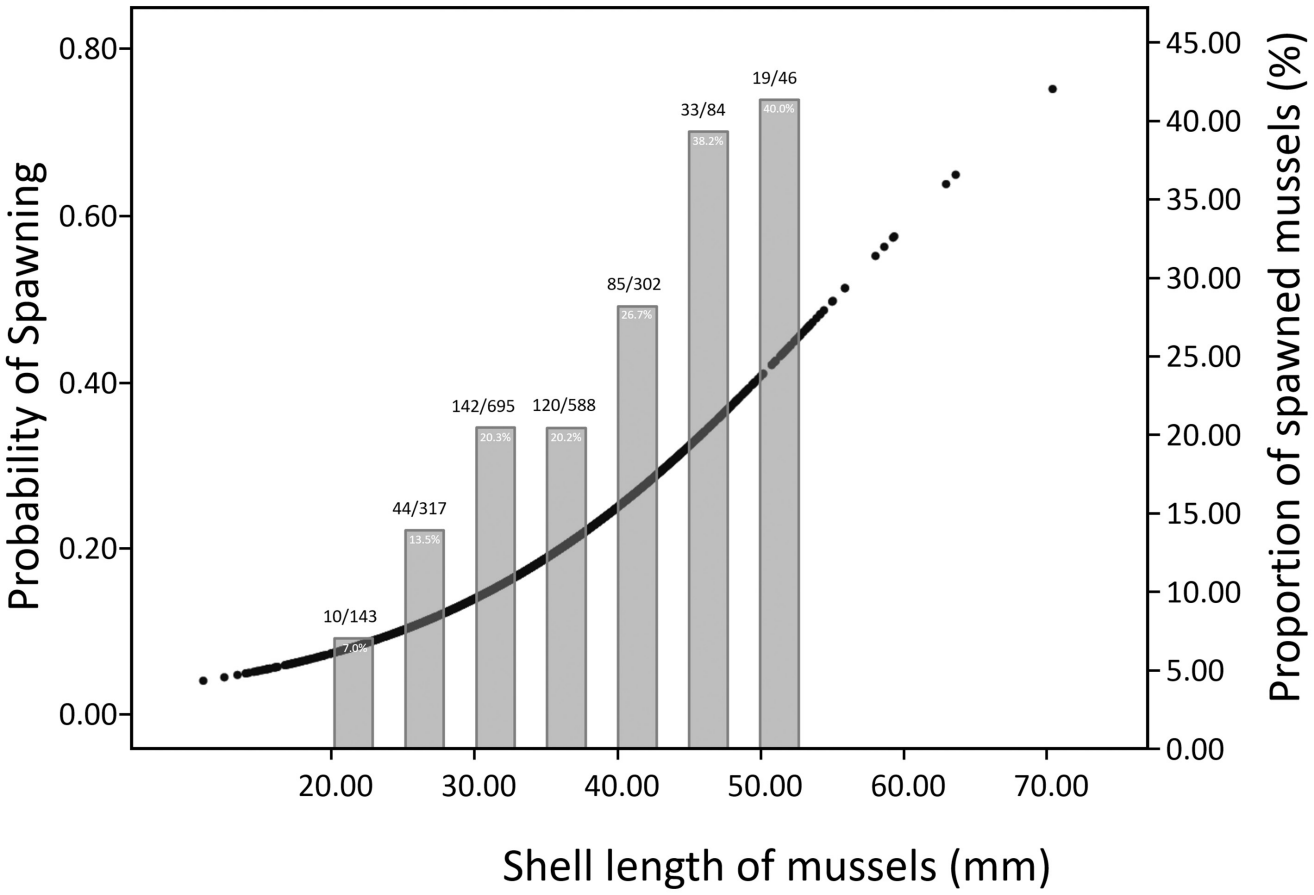
Proportion (%) of spawned mussels increases as shell length size classes increase. Size classes are divided by 5 mm interval in shell length. Numbers indicated above each bar show number of spawned mussels (left) and a total number of mussels collected (right). Black dots indicate the probability of spawning in mussels of different shell lengths [simple logistic regression; *B* = 1.075, 95% CI (1.059, 1.092), *p* < .001].

The spawning frequencies of entire bitterling fish communities were found to be significantly greater for larger mussel groups than small ones (Mann–Whitney *U* test, *p* < .001; Figure 4, Table 2). The mean shell length of the spawned mussels (mean ± SD, 38.07 ± 6.73 mm, *N* = 453) was 3.64 mm larger than that of unspawned mussels (34.43 ± 7.25 mm, *N* = 1814), and no mussels of <22 mm in shell length were spawned by bitterling fishes (Figure 4a). For each locality, the spawned mussel groups (29.81–45.89 mm) had larger mean shell length than unspawned ones (27.53–42.17 mm) except for the II locality, but only seven (YI, CH, HD, HI, GS, DY and GD) of the 17 comparisons were significant (Mann–Whitney *U* tests, *p* < .05; Figure 4b, Table 2). In addition, we found that percentage (%) of spawned mussels increases proportionally as increasing size classes (5 mm intervals) of the mussel groups (Figure 5). When the mussels were of >46 mm in shell length, the probability that would be spawned by bitterling fishes was >39% (Figure 5). The probability of spawning at host mussels was thus significantly higher in larger mussels than small ones [*B* = 1.075, 95% CI (1.059, 1.092), *p* < .001, Figure 5].

**TABLE 2:** Number of mussels collected and mean of mussel shell length (spawned or unspawned mussels), proportion of spawned mussels, number of bitterling species observed, number of mussel species observed, number of eggs/larvae, mean number of eggs/larvae per mussel and mussel density for each location.

Location	Number of mussels collected		Mean of mussel shell length		*p*‐Value	Proportion (%) of spawned mussels	Number of bitterling species	Number of mussel species	Number of eggs/ larvae	Mean number of eggs/larvae per mussel	Mussel density (m^2^)
	Spawned mussels^b^	Unspawned mussels^a^	Spawned mussels	Unspawned mussels							
YI	51	27	44.22 ± 7.29	30.42 ± 8.51	<.001	65.38	6	4	846	16.0	9.57
CH	11	134	45.89 ± 9.05	33.91 ± 11.06	.001	7.59	2	2	94	8.5	11.00
II	11	58	36.94 ± 4.28	37.27 ± 4.38	.731	15.94	1	1	46	4.2	3.57
GG	14	117	39.18 ± 4.06	36.44 ± 5.74	.064	10.69	1	1	45	3.2	6.00
HN	13	139	29.81 ± 2.58	29.70 ± 5.06	.893	8.55	1	1	29	2.2	6.57
HD	76	337	37.01 ± 3.68	35.03 ± 4.87	.002	18.40	4	1	348	4.6	1.29
HM	20	100	33.77 ± 2.62	33.47 ± 2.91	.561	16.67	1	1	52	2.6	10.57
HG	20	44	32.55 ± 4.59	29.19 ± 5.74	.087	31.25	2	1	288	14.4	–
HI	28	36	34.26 ± 5.31	30.11 ± 7.64	.021	43.75	1	1	118	4.2	8.29
GS	39	140	31.29 ± 4.90	27.53 ± 5.66	<.001	21.79	3	1	208	5.3	9.57
JG	63	240	38.06 ± 4.24	35.89 ± 6.69	.087	20.79	1	1	276	4.4	2.57
JJ	35	119	43.65 ± 6.16	42.17 ± 6.49	.460	22.73	2	1	91	2.6	9.00
PP	8	119	38.82 ± 3.45	38.16 ± 4.57	.787	6.30	2	2	27	3.4	7.71
YO	6	18	32.11 ± 3.33	29.83 ± 6.48	.343	25.00	1	1	13	2.2	–
DY	7	102	38.14 ± 2.64	34.61 ± 5.59	.046	6.42	1	1	28	4.0	6.86
DM	23	19	43.29 ± 6.53	39.87 ± 7.31	.060	54.76	2	1	86	3.7	10.14
GD	28	66	40.44 ± 5.78	33.31 ± 9.24	.001	29.79	3	3	346	11.9	9.89
Total	453	1814	38.07 ± 6.73	34.43 ± 7.25	<.001	19.98	7	4	2941	–	–

^a^ Number of mussels having no bitterling's eggs/larvae.

^b^ Number of mussels having bitterling's eggs/larvae.

**FIGURE 6:**
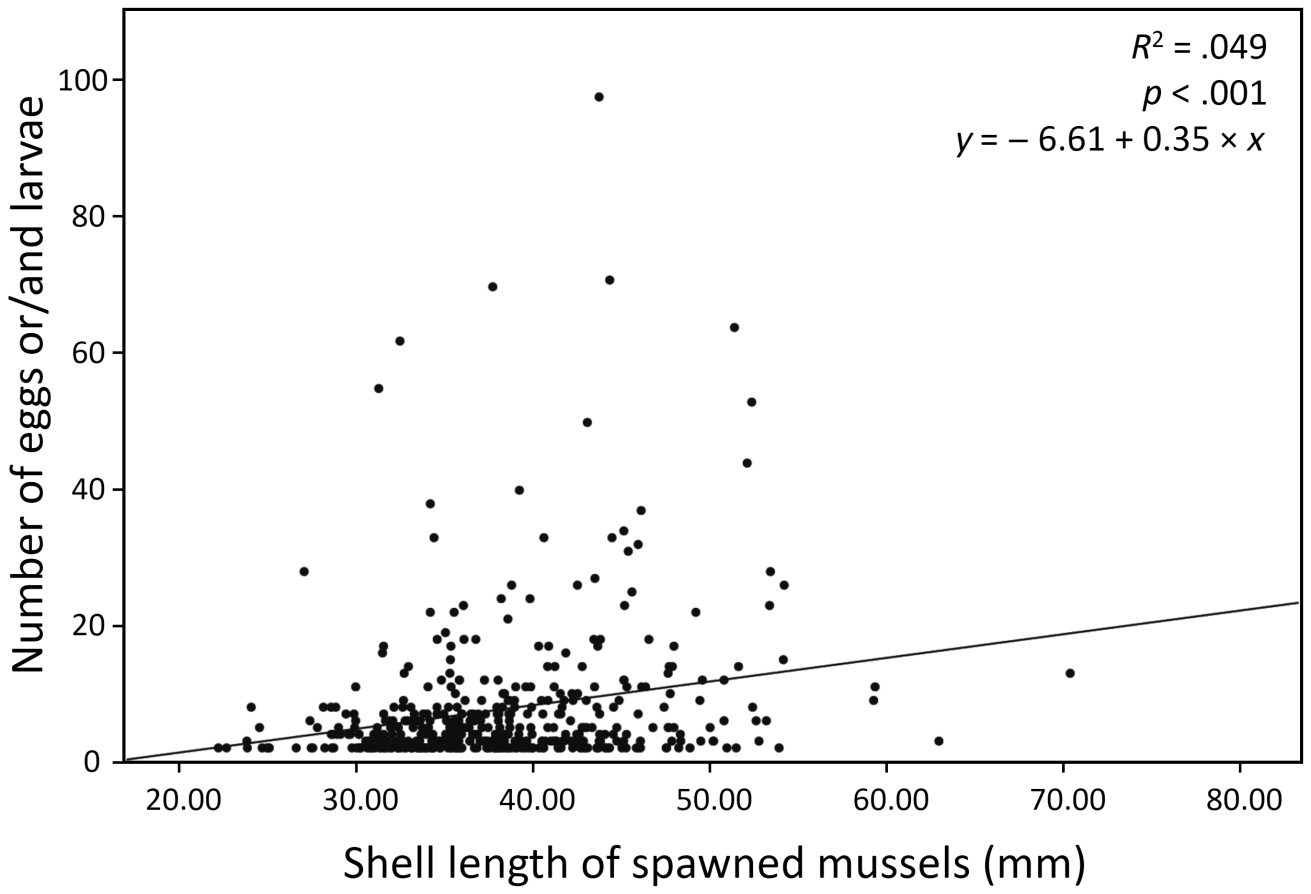
Linear regression analysis for relationship between shell length of the spawned mussels and number of eggs/larvae laid by bitterling fishes. Linear model, *Y*_number of eggs/larvae_ = −6.61 + 0.35 × *X*_mussel size_ (*R* ^2^ = .049, *p <* .001).

A significant positive linear relationship was observed between shell length of spawned mussels and number of eggs/larvae for the entire pooled communities (*R* ^2^ = .049, *F*_1,447_ = 22.786, *p* < .001; Figure 6). As mussel density was greater, proportion (%) of spawned mussels tended to increase, although not significant (*r* = .307, *p* = .265; Figure S2). These results might suggest that bitterling fishes tend to spawn aggregated, and more crowded mussel communities (Smith et al., 2006). Expectedly, a positive correlation was found between number of coexisting bitterling species and proportion (%) of spawned mussels (*r* = .543, *p* = .024; Table 2, Figure S3), although the samples were biased towards small number of one or two co‐occurring species.

### 3.2 Reciprocal transplant experiments

**FIGURE 7:**
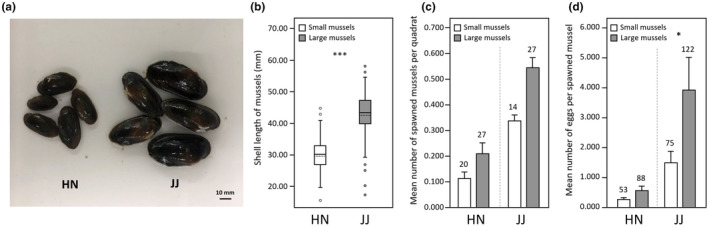
Results of reciprocal transplant experiments (see Figure 3). (a) Mussels collected on the two sites, including a small mussel habitat (HN) and a large mussel habitat (JJ). (b) Comparisons of the mean shell length (± SD) of mussels in each locality, the line within the box is the median; the dotted line within the box is the mean; the box marks the 25th and 75th percentiles; the whiskers mark the 10th and 90th percentiles and the circles represent outliers (HN: 29.71 ± 4.89 mm, JJ: 42.51 ± 6.43 mm). (c) Differences in mean (± SE) number of spawned mussels per quadrat between small (HN) and large (JJ) mussel habitats. (d) Differences in mean (± SE) number of eggs per spawned mussels between small (HN) and large (JJ) mussel habitats. Numbers above each bar indicate number of spawned mussels and also number of spawned eggs/larvae. Student's independent *t*‐tests; *p < .05, ***p < .001.

Two populations of large (mean shell length: JJ = 42.51 ± 6.43 mm) and small mussels (HN = 29.71 ± 4.89 mm) (Figure 7a,b) showed higher proportion of spawned frequencies in larger mussels in both native and non‐native (transplanted) hosts, but the results were only significant for comparisons of native large (JJ) versus transplanted small (HN) (Figure 7c,d, Table 3). Amount of eggs laid by bitterling fishes was also higher in larger mussels (Figure 7d, Table 3). In a small mussel habitat (HN), mean of spawned frequency per quadrat (mean ± *SE*, small‐mussel: 0.115 ± 0.025, large‐mussel: 0.212 ± 0.042; Student's *t*‐test; *p* = .053) and mean number of eggs laid per mussel (small‐mussel: 0.316 ± 0.084, large‐mussel: 0.656 ± 0.197; Student's *t*‐test; *p* = .088) were higher for the transplanted larger mussel groups, although not significant (Figure 7c,d, Table 3). Similarly, in a large mussel habitat (JJ), mean of spawned frequency per quadrat (small‐mussel: 0.337 ± 0.087, large‐mussel: 0.546 ± 0.115; Student's *t*‐test; *p* = .158) and mean number of eggs laid per mussel (small‐mussel: 1.500 ± 0.388, large‐mussel: 3.936 ± 1.086; Student's *t*‐test; *p* = .015) were higher for the native larger mussel groups, while only amount of eggs was significant (Figure 7c,d, Table 3). Overall, all these results showed trends of higher spawning frequencies and amount of eggs in larger mussels even in unfamiliar (non‐native) hosts from a foreign place.

**TABLE 3:** Results of reciprocal transplant experiments between two localities, including a small mussel habitat of HN and a large mussel habitat of JJ (Figures 3 and 7).

Population	Mussel type	Proportion of spawned mussels (number of spawned mussels/total number of mussels)	Mean number of spawned mussels per quadrat (± SE)	*p*‐Value	Number of spawned eggs/larvae	Mean number of spawned eggs/larvae per mussel (± SE)	*p*‐Value
Small mussel habitat (HN)	Small mussels (native)	20/169	0.115 ± 0.025	.053	53	0.316 ± 0.084	.088
	Large mussels (transplanted)	27/134	0.212 ± 0.042		88	0.656 ± 0.197	
Large mussel habitat (JJ)	Small mussels (transplanted)	14/50	0.337 ± 0.087	.158	75	1.500 ± 0.388	.015
	Large mussels (native)	21/31	0.546 ± 0.115		122	3.936 ± 1.086	

*Note*: Mussel type (native or non‐native [transplanted]), proportion of spawned mussels, mean number of spawned mussels per quadrat, number of spawned eggs/larvae and mean number of spawned eggs/larvae per mussel in the transplanted experiments. Statistical significance was tested using Student's independent *t*‐tests.

### 3.3 Differences in spawning strategies among bitterling species

**FIGURE 8:**
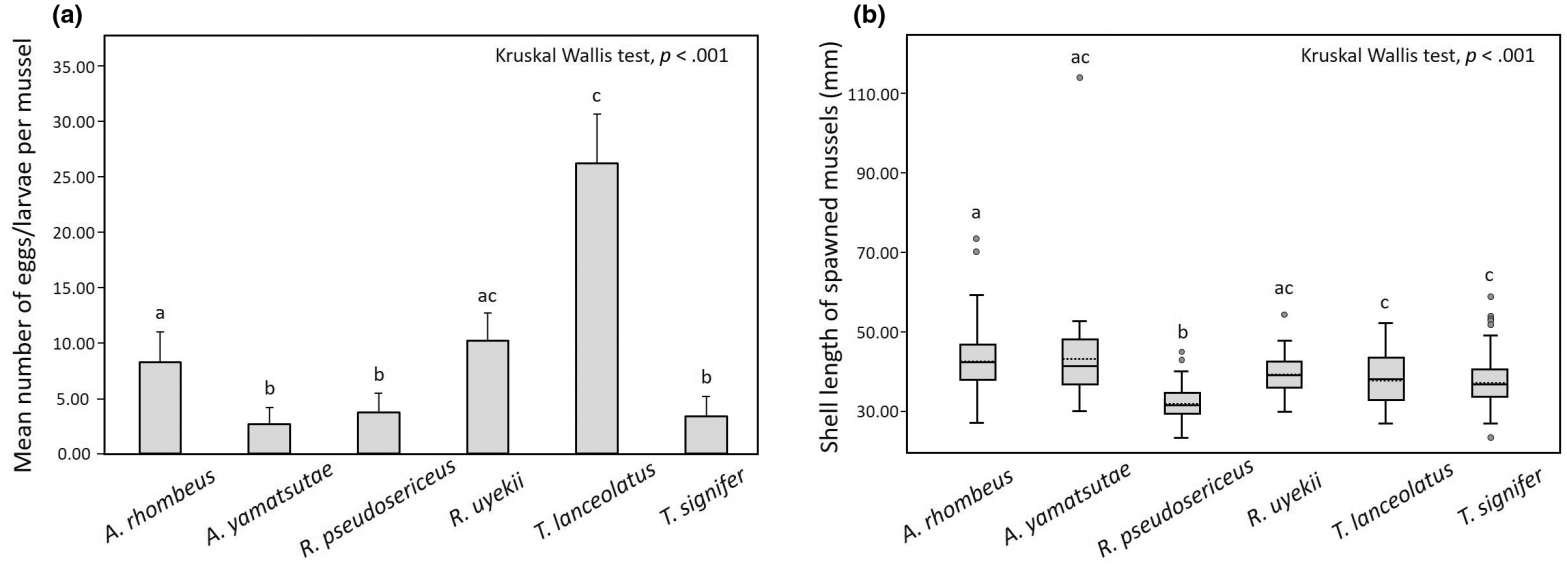
(a) Differences in mean number (± SE) of eggs/larvae among the six Acheilognathinae bitterling fish species. (b) Differences in mean shell length (± SD) of spawned mussels among the six species. (b) The solid line within the box is the median; the dotted line within the box is the mean; the box marks the 25th and 75th percentiles; the whiskers mark the 10th and 90th percentiles and the circles represent outliers. Statistical analyses were performed using Kruskal–Wallis tests. Different lowercase letters above bars or boxes show significantly different groups as determined by Dunn's multiple comparison tests; values with the same letters are not significant (Table S2).

We found significant differences in mean number (± SE) of eggs/larvae per mussel among the six bitterling species (Kruskal–Wallis test, *H* = 124.68, *df* = 5, *p* < .001; Figure 8a, Table 4). Two species of *R. ocellatus ocellatus* and *R. notatus* were excluded from this analysis owing to a small number of spawned mussels for *R. ocellatus ocellatus* (*N* = 2, number of eggs/larvae per mussel: 16.00 ± 4.61) or no eggs/larvae detected for *R. notatus*. *Tanakia lanceolatus* spawned a significantly higher mean of 26.38 eggs/larvae per mussel than four other species (Dunn's tests, *T. lanceolatus* vs. *A. rhombeus*: *p* = .001; vs. *A. yamatsutae*: *p* < .001; vs. *R. pseudosericeus*: *p* < .001; vs. *T. signifer*: *p* < .001; Table S2). While *A. rhombeus* (8.29 ± 2.78) and *R. uyekii* (10.23 ± 2.51) showed an intermediate mean number of eggs/larvae per mussel, *A. yamatsutae* (2.69 ± 2.21), *R. pseudosericeus* (3.72 ± 1.79) and *T. signifer* (3.40 ± 1.82) showed a significantly smaller number of offspring per mussel (Table S2).

**TABLE 4:** Results of species‐level spawning patterns for each location.

Location	Species identified	Mean shell length of spawned mussels (mm)	Number of mussels having eggs/larvae	A total number of eggs/larvae found	Mean number of eggs/larvae per mussel	Number of eggs/larvae found in inner gills	Number of eggs/larvae found in outer gills
YI	*A. rhombeus*	44.27	36	359	9.97	316	43
	*A. yamatsutae*	82.90	2	4	2.00	2	2
	*R. ocellatus ocellatus*	88.19	1	31	31.00	31	–
	*R. uyekii*	43.53	7	47	6.71	30	17
	*T. lanceolatus*	41.57	15	403	26.87	55	348
	*T. signifer*	44.18	2	2	1.00	1	1
CH	*A. rhombeus*	48.20	4	62	15.50	61	1
	*T. signifer*	46.39	7	32	4.57	13	19
II	*T. signifer*	36.94	11	46	4.18	–	46
GG	*T. signifer*	39.18	14	45	3.21	4	41
HN	*T. signifer*	29.85	11	29	2.64	–	–
HD	*A. yamatsutae*	37.46	15	37	2.47	–	–
	*R. ocellatus ocellatus*	30.13	1	1	1.00	–	–
	*R. uyekii*	37.38	15	178	11.87	–	–
	*T. signifer*	37.07	43	132	3.07	–	–
HM	*T. signifer*	33.77	20	52	2.60	–	52
HG	*R. pseudosericeus*	31.48	13	43	3.31	7	36
	*T. lanceolatus*	34.82	8	245	30.63	31	214
HI	*R. pseudosericeus*	34.26	28	118	4.21	31	87
GS	*A. rhombeus*	36.27	10	40	4.00	40	–
	*R. pseudosericeus*	30.70	27	92	3.41	32	60
	*T. lanceolatus*	34.25	6	76	12.67	7	69
JG	*T. signifer*	38.06	63	276	4.38	–	7
JJ	*A. yamatsutae*	47.56	11	30	2.73	19	8
	*T. signifer*	42.89	24	61	2.54	4	23
PP	*A. yamatsutae*	38.43	7	25	3.57	25	–
	*T. signifer*	41.61	1	2	2.00	–	2
YO	*T. signifer*	32.11	6	13	2.17	4	9
DY	*T. signifer*	38.14	7	28	4.00	23	5
DM	*A. rhombeus*	45.64	16	77	4.81	74	3
	*T. signifer*	37.05	5	9	1.80	–	9
GD	*A. rhombeus*	41.03	26	225	8.65	214	11
	*A. yamatsutae*	34.51	1	1	1.00	1	–
	*T. lanceolatus*	36.11	3	120	40.00	–	120
Total	*A. rhombeus*	42.90	92	763	8.29	705	58
	*A. yamatsutae*	43.18	36	97	2.69	47	10
	*R. ocellatus ocellatus*	59.16	2	32	16.00	31	–
	*R. pseudosericeus*	32.31	68	253	3.72	70	183
	*R. uyekii*	39.34	22	225	10.23	30	17
	*T. lanceolatus*	38.00	32	844	26.38	93	751
	*T. signifer*	37.75	214	727	3.40	49	214

*Note*: Species identification was undertaken using our developed restriction fragment length polymorphism (RFLP) marker (Figure S1, Table 1). Mean shell length of spawned mussels, number of mussels having eggs/larvae, a total number of eggs/larvae found, mean number of eggs/larvae per mussel, number of eggs/larvae found in inner gills and number of eggs/larvae found in outer gills.

The mean shell length (± SD) of spawned mussels significantly varied among the six bitterling species (Kruskal–Wallis test: *H* = 114.61, *df* = 5, *p* < .001; Figure 8b, Table 4). *Rhodeus pseudosericeus* spawned in mussels of significantly (5.44–10.86 mm in shell length) smaller shell lengths compared to the other five species (Dunn's tests, *R. psudosericeus* vs. *A. rhombeus*: *p* < .001; vs. *A. yamatsutae*: *p* < .001; vs. *R. uyekii*: *p* < .001; vs. *T. lanceolatus*: *p* < .001; vs. *T. signifer*: *p* < .001; Table S2). *Acheilognathus rhombeus* (42.90 ± 6.79 mm), *A. yamatsutae* (43.18 ± 14.06 mm) and *R. uyekii* (39.34 ± 5.56 mm) spawned in relatively large mussels, whereas *T. lanceolatus* (38.00 ± 6.76 mm) and *T. signifer* (37.75 ± 5.32 mm) intermediate sized mussels (Table S2). Mean of *R. ocellatus ocellatus*' spawned mussel length was 59.16 ± 41.05 mm (*N* = 2), although this species excluded from statistical analysis.

**FIGURE 9:**
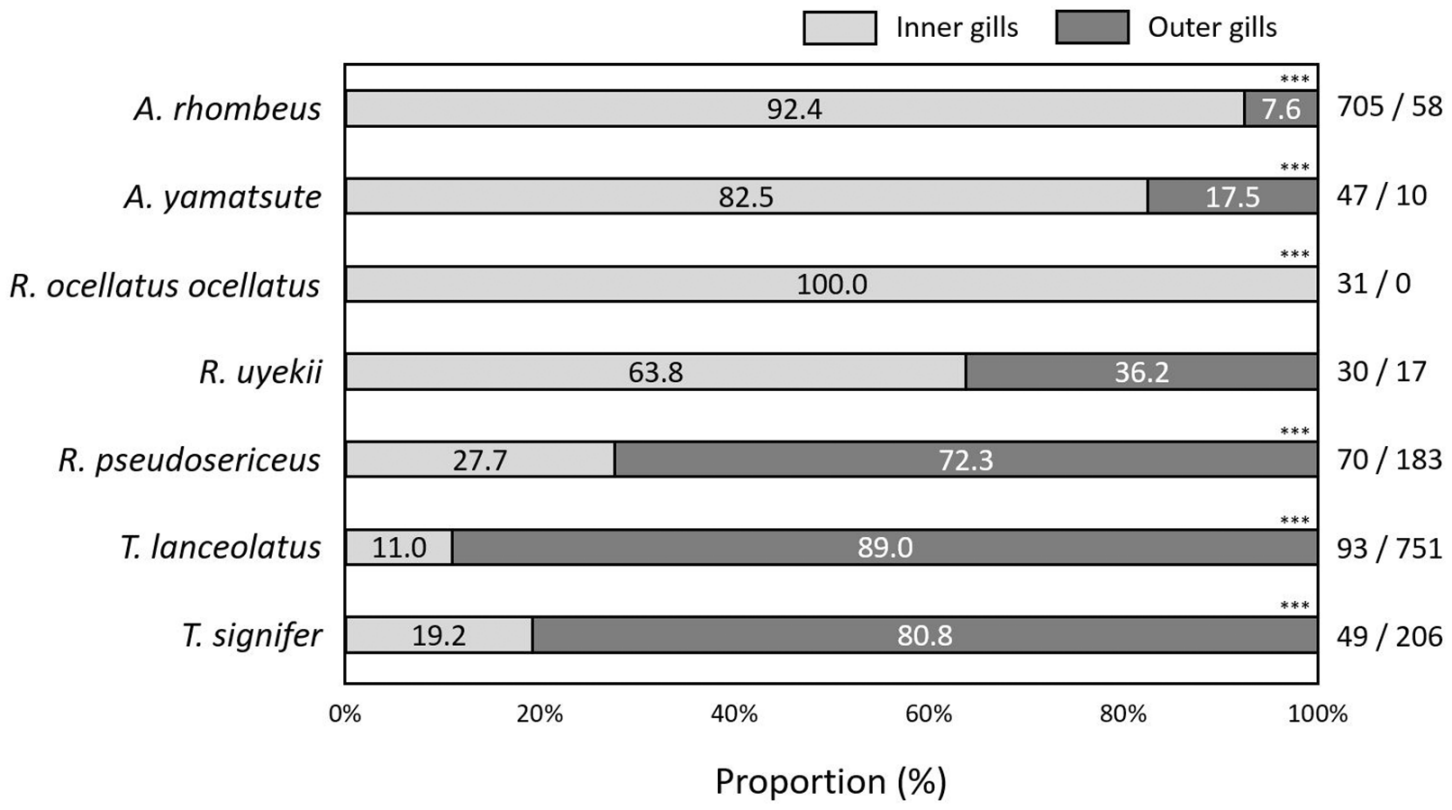
Proportion of spawning positions of seven Acheilognathinae species as estimated from number of eggs/larvae observed in the inner and outer parts of the gills inside the mussels. Numbers shown at the right indicate number of eggs/larvae detected (inner gills [left]/outer [right]). The observed proportions of the gill positions of the seven bitterlings were all significantly different from an expected ratio of inner: outer gills = 1:1 (***χ ^2^ tests, *p* < .001).

The use of gill positions for spawning was non‐random and differed among the six bitterling species except for *R. uyekii* (*p* = .058), meaning that *A. rhombeus* (*χ* ^2^ tests, *p* < .001), *A. yamatsutae* (*p* < .001) and *R. ocellatus ocellatus* (*p* < .001) spawned more in the inner gills, whereas *R. pseudosericeus* (*p* < .001), *T. lanceolatus* (*p* < .001) and *T. signifer* (*p* < .001) higher in the outer gills. The observed proportions of the gill positions of these six bitterlings (except for *R. uyekii*) were all significantly different from an expected ratio of inner: outer gills = 1:1 (Figure 9).

### 3.4 Morphological adaptation of female *T. signifer* to the host size

**FIGURE 10:**
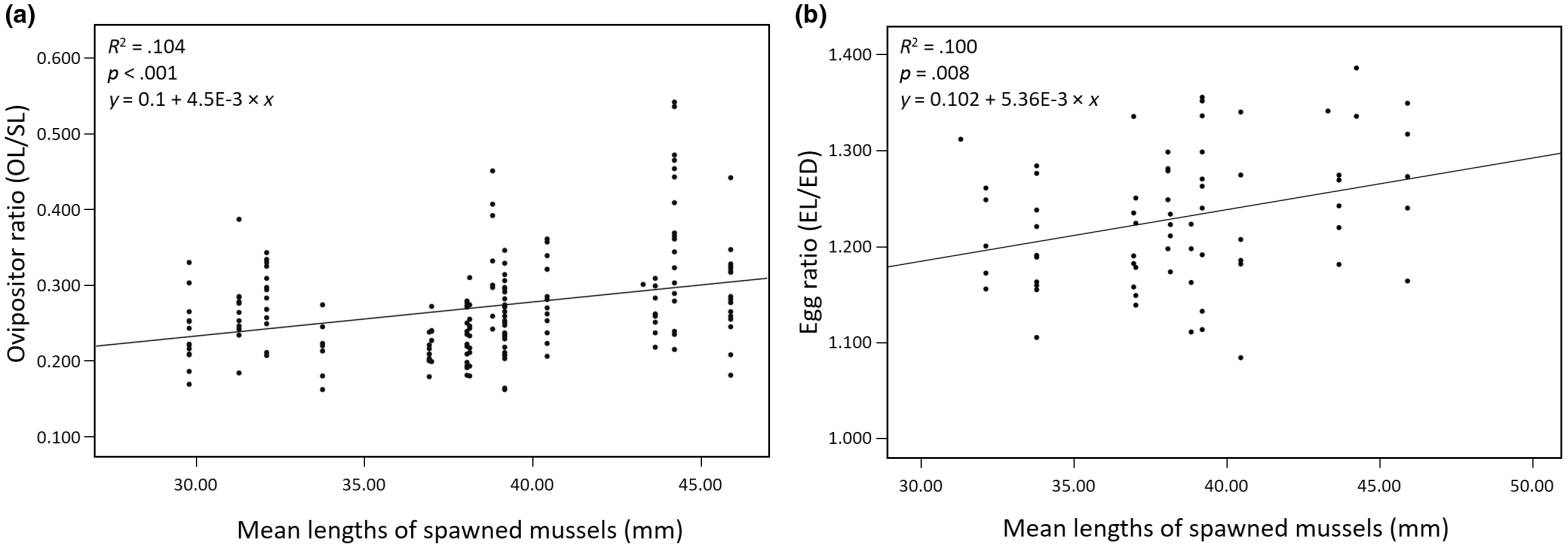
Results of reproductive traits of female *Tanakia signifer* in relation to the mussel shell size. (a) Linear regression analysis for relationship between mean length of spawned mussels and ovipositor ratio (ovipositor length/standard length). Linear model, *Y*_ovipositor ratio_ = 0.1 + 4.5E‐3 × *X*_mussel size_ (*R* ^2^ = .104, *p* < .001). (b) Linear regression analysis for relationship between mean length of spawned mussels and egg ratio (egg length/egg diameter). Linear model, *Y*_egg ratio_ = 0.102 + 5.36E‐3 × *X*_mussel size_ (*R* ^2^ = .100, *p* = .008).

A significant positive linear relationship was found between mean length of spawned mussels and ovipositor ratio (OL/SL) of *T. signifer* females (*R* ^2^ = .104, *F*_1,180_ = 20.902, *p* < .001; Figure 10a, Table 5), suggesting that *T. signifer* females evolved a longer ovipositor in larger mussel host environments. Also, egg ratio (EL/ED) was significantly positively associated with mean length of spawned mussels (*R* ^2^ = .100, *F*_1,66_ = 7.371, *p* = .008; Figure 10b, Table 5), indicating that *T. signifer* may have evolved more elongated eggs in larger mussels.

**TABLE 5:** Reproductive traits (e.g. ovipositor length and egg shape) of *Tanakia signifer* females at 15 localities in the Han River basin.

Location	Mean shell length of spawned mussels	Number of females analysed (for ovipositor length/egg shape)	Number of extracted eggs from females	Female standard length (SL) mm	Ovipositor length (OL) mm	Ovipositor ratio (OL/SL)	Egg length (EL) mm	Egg diameter (ED) mm	Egg ratio (EL/ED)
YI	44.22 ± 7.29	18/2	20	43.41 ± 8.15	15.46 ± 3.16	0.37 ± 0.10	1.89 ± 0.07	1.40 ± 0.04	1.36 ± 0.05
CH	45.89 ± 9.05	17/5	45	48.23 ± 8.33	13.61 ± 2.30	0.29 ± 0.06	1.83 ± 0.10	1.45 ± 0.04	1.26 ± 0.08
II	36.94 ± 4.28	10/5	49	60.64 ± 3.98	12.44 ± 0.93	0.21 ± 0.02	1.74 ± 0.11	1.44 ± 0.07	1.22 ± 0.08
GG	39.18 ± 4.06	30/5	47	57.04 ± 5.28	14.27 ± 2.00	0.25 ± 0.04	1.91 ± 0.12	1.49 ± 0.03	1.28 ± 0.08
HN	29.81 ± 2.58	13/5	39	53.64 ± 5.35	12.57 ± 2.74	0.23 ± 0.04	1.78 ± 0.12	1.53 ± 0.08	1.16 ± 0.06
HD	37.01 ± 3.68	5/5	46	46.82 ± 7.78	10.86 ± 1.79	0.23 ± 0.03	1.56 ± 0.13	1.31 ± 0.10	1.19 ± 0.06
HM	33.77 ± 2.62	7/5	45	51.35 ± 6.06	10.85 ± 1.02	0.21 ± 0.04	1.62 ± 0.08	1.34 ± 0.09	1.22 ± 0.06
GS	31.29 ± 4.9	12/6	54	50.46 ± 6.38	13.19 ± 1.40	0.27 ± 0.05	1.77 ± 0.12	1.42 ± 0.06	1.25 ± 0.10
JG	38.06 ± 4.24	15/5	33	56.46 ± 3.66	13.06 ± 1.81	0.23 ± 0.04	1.83 ± 0.08	1.46 ± 0.04	1.25 ± 0.06
JJ	43.65 ± 6.16	9/5	38	53.89 ± 7.29	14.02 ± 1.63	0.26 ± 0.03	1.79 ± 0.13	1.44 ± 0.07	1.24 ± 0.06
PP	38.82 ± 3.45	8/4	26	46.16 ± 5.74	15.31 ± 3.92	0.33 ± 0.07	1.57 ± 0.08	1.34 ± 0.09	1.18 ± 0.06
YO	32.11 ± 3.33	14/5	45	54.38 ± 5.72	15.27 ± 2.51	0.28 ± 0.04	1.70 ± 0.14	1.42 ± 0.07	1.20 ± 0.06
DY	38.14 ± 2.64	10/5	47	57.87 ± 6.5	13.39 ± 1.56	0.23 ± 0.04	1.75 ± 0.09	1.42 ± 0.05	1.23 ± 0.07
DM	43.29 ± 6.53	1/−	–	48.49	14.52	0.30	–	–	–
GD	40.44 ± 5.78	13/6	49	46.69 ± 6.68	13.16 ± 2.46	0.28 ± 0.05	1.72 ± 0.12	1.44 ± 0.11	1.20 ± 0.09
Total	38.07 ± 6.73	182/68	583	52.26 ± 7.90	13.69 ± 2.49	0.27 ± 0.07	1.75 ± 0.15	1.42 ± 0.09	1.23 ± 0.08

## 4 DISCUSSION

### 4.1 Spawning patterns of the bitterling fishes in Korea

The results of our study show that bitterling fishes from the Han River in South Korea more frequently use larger mussels as their hosts for spawning and nursing at entire bitterling and mussel communities as well as at most locality. The observed spawning preference, and higher spawning frequency and magnitude in larger mussels are further supported by the results of our reciprocal transplantation experiments that bitterlings tend to more often reproduce in larger mussels with more eggs, although the hosts are unfamiliar to the fishes, albeit not significant (Figure 7). We further show that the probability of spawning proportionally increases with mussel size (Figure 5). The findings of bitterling's larger mussel preferences as a spawning substrate were consistent with previous studies of Japanese striped bitterling species, *Acheilognathus cyanostigma* (Kitamura, 2006) and also of *T. signifer*, one of the study species, occurring in Korea (Kim et al., 2014). The ecological advantages of the use of larger mussels may involve fitness gains through enhanced offspring survival. A previous study demonstrated a positive association of the mussel size with respiration rates (Trigos et al., 2015). Female bitterlings are known to evaluate the oxygen contents of the water nearby the exhalant siphon of the mussels by using a head‐down posture and then select their target hosts for spawning (Smith et al., 2001, 2004). Larger mussels may supply a higher amount of oxygen for the bitterling's offspring, which could drive adaptive evolution by natural selection acting on the offspring survival. This means that offspring reared in larger hosts probably survive better or grow faster than those in small ones. Nevertheless, there would be a positive correlation between mussel respiration rate and egg ejection rate, which means that larger mussels could have detrimental effects on bitterling's offspring survival through higher ejection rates of eggs (Reichard, Liu, & Smith, 2007). Although costs imposed by bitterling on host mussels may favour the evolution of egg ejection behaviour as a host defence, there is no empirical evidence supporting the hypothesis that egg ejection behaviour evolved in mussels responding to the bitterling's brood parasitism (Mills & Reynolds, 2003). The evolution of behavioural trait of the ejection response in mussels has been suggested as an evolutionary lag in the relationship between bitterling and mussels (Reichard, Przybylski, et al., 2007). Asian mussels, *Anodonta woodiana*, which has long coexisted with several bitterling species for at least 16 million years, evolved more pronounced ejection behaviour than European mussels, which have a relatively short history of coexistence (Reichard, Przybylski, et al., 2007). In a recent study on the host preference of bitterling, *Acheilognathus typus* with respect to the mussel (*Sinanodonta lauta*) size in an experimental enclosure pond, 27 out of 44 bitterling pairs examined selected larger mussels (over 110 mm) for their spawning (Fujimoto et al., 2022). However, the study also found that egg ejection more frequently occurred in larger mussels (Fujimoto et al., 2022). Our results indicate that spawning in small hosts of <22 mm in shell length appears to be detrimental and may thus be eliminated by negative selection. In the proportion of spawned mussels grouped by mussel size (5 mm interval), the spawning ratio and spawning probability were proportionally higher as mussel size increases. These findings clearly suggest that bitterlings might have evolved choosing large‐sized mussels as a symbiosis for spawning as they gain the fitness benefits of increasing offspring survival via high oxygenation for their eggs, although there may be a risk of egg ejection. Nevertheless, the intensity of selection against choosing larger mussels because of egg ejection behaviour needs to be investigated more thoroughly. Overall, the results of our study suggest that ‘larger’ sized mussels are probably an optimal for the evolution of host preference in bitterlings, considering fitness benefits and fitness costs (Parker & Smith, 1990).

We find a tendency that the mussel density is positively correlated with the proportion of spawned mussels, although not significant (Figure S2). A study of European bitterling fish species, *Rhodeus sericeus* found a positive correlation between mussel density and number of eggs spawned (Reichard et al., 2004). These results would be associated with the courtship behaviour of males to guard several mussels in their territory (often defend only a single mussel) and to attract females (Schaumburg, 1989). Also, there was a positive correlation between the number of coexisting bitterling species and the proportion of spawned mussels (Figure S3). This suggests that if mussel populations decrease in a habitat where several bitterling species coexist, hybridization and genetic introgression may occur due to extreme resource competition (Hata et al., 2019, 2021; Uemura et al., 2018). In our results, two bitterling species (*A. rhombeus*, *A. yamatsutae*, *N* = 1; *A. rhombeus, R. pseudosericeus*, *N* = 4; *A. rhombeus*, *R. uyekii*, *N* = 4; *A. rhombeus*, *T. lanceolatus, N* = 13; *A. rhombeus*, *T. signifer*, *N* = 2; *A. yamatsutae*, *T. signifer*, *N* = 4; *R. pseudosericeus*, *T. lanceolatus*, *N* = 5; *R. uyekii*, *T. lanceolatus*, *N* = 1) frequently spawned in the same mussel individuals simultaneously. The most frequently observed species spawning with other bitterling fishes in the same hosts was *A. rhombeus*, which had spawned in autumn of the previous year before the other bitterling species reproduced in spring of 2019. However, we could not observe that more than two fish species spawned in the same mussel individuals in the Han River (Choi & Lee, 2019). However, in our more recent investigation, it was found that three bitterling species sometimes spawn in the same mussels, particularly when the mussel density is extremely low (e.g. mussel density = 1.14 per 1 × 1 m^2^) (Seo et al., 2023). A recent study observed a natural hybrid between a female *T. lanceolata* and a male *R. peudosericeus* using a combined analysis of maternally inherited mtDNA cyt *b* and paternally inherited nuclear DNA (nuDNA) recombination activating gene 1 (*rag* 1) in a river flowing into the West Sea in Korea (Kim et al., 2021; Kim, Yun, et al., 2015). Hybridization can lead to direct or indirect damaging effects on endemic species or locally adapted populations through introgression (Allendorf et al., 2001; Scribner et al., 2000). Therefore, for the conservation of bitterling fish populations, it will be necessary to maintain or recover the number of host mussel populations by considering the spawning preferences of each species.

### 4.2 Differences in reproductive outputs, preferred host sizes and spawning slots among bitterling species

Our study shows significant differences in mean number of eggs/larvae per mussel among the six bitterling species, suggesting that different reproductive strategies (or fecundity) evolve in Korean bitterling fishes. A strategy of high number of eggs might have evolved in *T. lanceolatus*, *R. uyekii* and *A. rhombeus*, whereas relatively lower number of offspring in *A. yamatsutae*, *R. pseudosericeus* and *T. signifer* (Figure 8a) (Aldridge, 1999). *Tanakia lanceolatus* laid the greatest mean number of 26.38 eggs per mussel and *R. uyekii* spawned the second largest mean number of 10.23 eggs per mussel. These two high‐fecundity species are known to have sticky eggs (fusiform) and thus yield an egg clutch aggregated inside the gills of mussels (Kim, Ko, et al., 2015). *Rhodeus ocellatus ocellatus* spawned 16.00 eggs per mussel (bulb like), although the number of spawned mussels observed was very small (*N* = 2). Therefore, it's spawning features could not be precisely determined. An autumn spawning bitterling, *A. rhombeus* laid on average 8.29 eggs per mussel. This species is known to spawn in a fall season of September–November, and the spawned larvae undergo diapause inside the mussel gills over the winter and then restart and complete developmental processes in the upcoming spring (Kim et al., 2018). The eggs/larvae of *A. rhombeus* observed in this study were at a developmental stage just after diapause. *Acheilognathus rhombeus* might have an advantage of using empty mussels (with no spawned eggs/larvae) in autumn without any interspecific competition (i.e. ‘preemption’), and this may account for a relatively high proportion of 25.9% (*A. rhombeus* eggs/larvae: *N* = 763, total eggs/larvae: *N* = 2941) in total numbers of eggs/larvae analysed. The remaining three species, *A. yamatsutae*, *R. pseudosericeus* and *T. signifer*, were identified as mean number of 2.69, 3.72 and 3.40 eggs per mussel respectively. In a previous study, eggs of the genus *Acheilognathus* are ovoid‐shaped and smaller in size than those of the genus *Rhodeus*, and several batches of eggs would be laid in the same mussels (Aldridge, 1999). *Rhodeus pseudosericeus* and *T. signifer* have been listed as an endangered species. In particular, *R. pseudosericeus* exploits significantly smaller mussels for spawning (Figure 8b). These results suggest that sustaining or repopulating relatively smaller mussels in the habitats would be key for the effective conservation and management for *R. pseudosericeus*.

The spawning positions within the gills of the mussels differ by species, meaning that *A. rhombeus*, *A. yamatsutae* and *R. ocellatus ocellatus* prefer the inner gills as their spawning slots, whereas *R. pseudosericeus*, *T. lanceolatus* and *T. signifer* the outer gills. Yet, *R. uyekii* shows a borderline significance in the spawning positions between inner and outer gills. These results may suggest species‐specific preference for particular inner/outer spawning positions, or resource/niche partitioning for the spawning space among the species (Kitamura, 2007). The former hypothesis could mean ‘innate’ interspecific difference in the spawning positions while the latter would account for the evolutionary outcomes from strong interspecific competition by natural selection. Different bitterling species sharing the same host individuals could provide some evidence for interspecific resource competition. However, the observation that the same three bitterling species (*A. yamatsutae*, *R. uyekii* and *T. latimarginata*) occurring at geographically separated different rivers showed the same preferences for the particular spawning positions could provide evidence supporting the ‘innate’ species specific preference hypothesis (Seo et al., 2023). Thirty‐four mussel individuals (7.5%) incubated two bitterling species' progenies. In the case of *A. rhombeus*, the inner gills were preferred despite in the absence of other competing bitterling species. Nevertheless, given the egg ejection of the mussels could not be considered in this study, additional research in the laboratory condition will be required to more precisely determine the preference for the mussel gill position as spawning slots.

### 4.3 Morphological adaptation of female's reproductive traits to the host size

We find a positive association of morphology in female's reproductive traits of *T. signifer*, such as ovipositor length and egg shape, with the host mussel's shell length (Figure 10). These results suggest that morphological characteristics of the spawning phenotypes in *T. signifer* females may have evolved higher fitness responding to host environments and diverged in different host mussel conditions (Kitamura et al., 2012). The findings indicate that *T. signifer* populations that exploit larger mussels have evolved longer ovipositors and more elongated eggs as a result of morphological adaptions, where females with longer ovipositors and lengthened eggs enjoy higher fitness in large mussel environments. A previous study of the Japanese bitterling species, *Acheilognathus tabira* also found the similar patterns of a correlation of ovipositor length and egg shape with differences in host mussel species (family Unionidae) that differ in body size (Kitamura et al., 2012). Significant differences in morphological features in female's reproductive characteristics (ovipositor length and egg shape) in *T. signifer* among the mussel populations of different shell sizes may result from effects of natural selection, leading to morphological adaptation to different host environments (Schluter, 2000). It would also be possible that morphological differences in reproductive traits of *T. signifer* females among the mussel populations may be the result of phenotypic plasticity. To show the differences in morphology have a genetic basis, rearing these bitterling females in a common garden environment would be required. Given there is considerable neutral genetic divergence among *T. signifer* populations at the study sites (Choi and Lee, unpublished data), the former hypothesis of ‘the genetic adaptation of morphology in ovipositor length and egg shape by natural selection’ would be more likely. However, the neutral genetic divergence is not directly related to the development of the female's reproductive phenotypes.

The findings of our study will help to advance our understanding of the spawning patterns and reproductive ecology of an idiosyncratic mussel‐symbiotic bitterling fishes in regard to the host size from a major river system in Korea. The results of this study will also provide an insight into the long‐standing evolutionary question of how and why the symbiotic interaction between bitterlings and mussels may evolve. We will further identify the mechanisms underlying the co‐evolutionary processes by testing three possible hypotheses, such as mutualism, commensalism and parasitism. Furthermore, this study will inform on the development of effective conservation and management strategy for the endangered bitterling fishes in Korea.

## AUTHOR CONTRIBUTIONS

**Hee‐kyu Choi:** Conceptualization (equal); data curation (lead); formal analysis (lead); funding acquisition (supporting); investigation (lead); methodology (equal); project administration (supporting); visualization (equal); writing – original draft (lead). **Hyuk Je Lee:** Conceptualization (equal); data curation (supporting); formal analysis (supporting); funding acquisition (lead); investigation (supporting); methodology (supporting); project administration (lead); supervision (lead); writing – review and editing (lead).

## CONFLICT OF INTEREST STATEMENT

The authors declare that they have no conflict of interest.

## Supporting information


Figure S1.

Figure S2.

Figure S3.

Table S1.

Table S2.

## Data Availability

The datasets generated during the present study are available from the corresponding author upon reasonable request. Some data are available in Tables 1, 2, 3, 4 and supplementary material (Appendices) has been included as part of Supporting Information.
