# Supplementary figures and images for: Host size matters for reproduction: Evolution of spawning preference and female reproductive phenotypes in mussel‐symbiotic freshwater bitterling fishes
Source: Ecol Evol. 2024 Mar 11;14(3):e11142. doi: 10.1002/ece3.11142 (PMC10927361; doi:10.1002/ece3.11142)

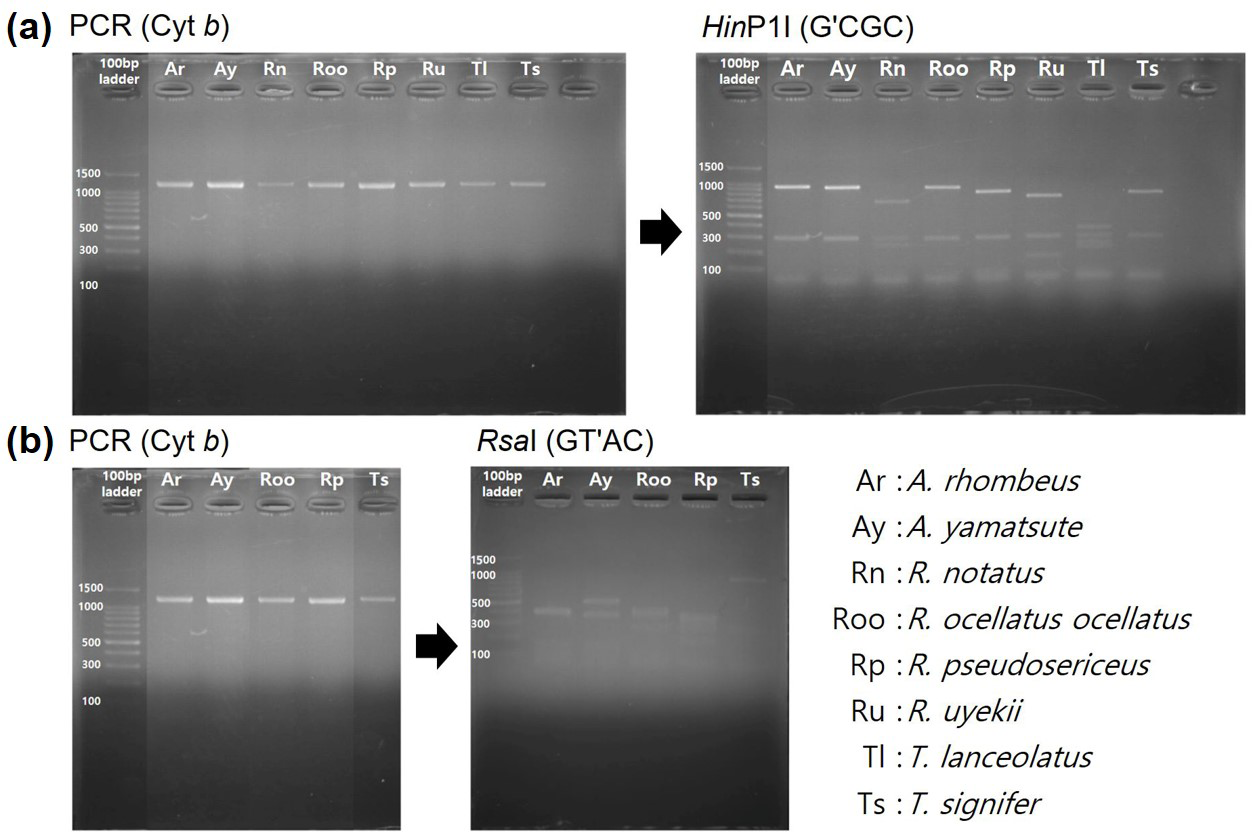

Supplement: Supplementary file 1 — Figure S1. [file ECE3-14-e11142-s005.tif]

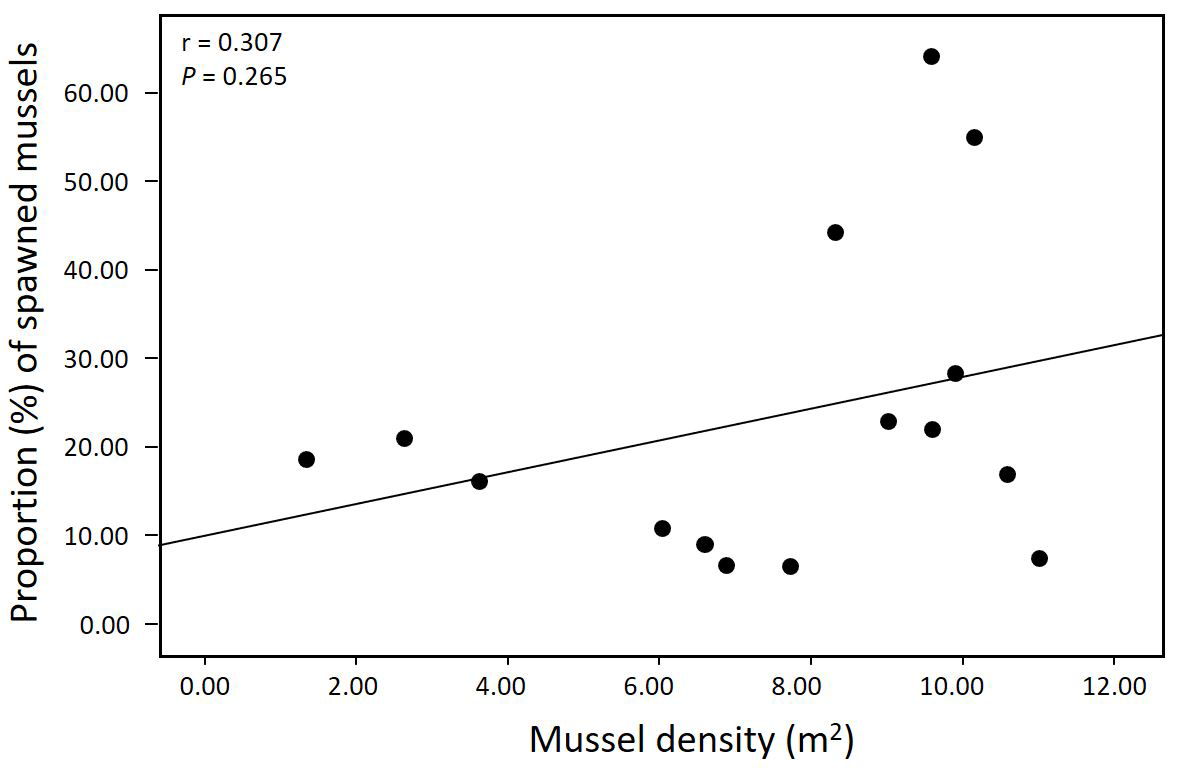

Supplement: Supplementary file 2 — Figure S2. [file ECE3-14-e11142-s001.tif]

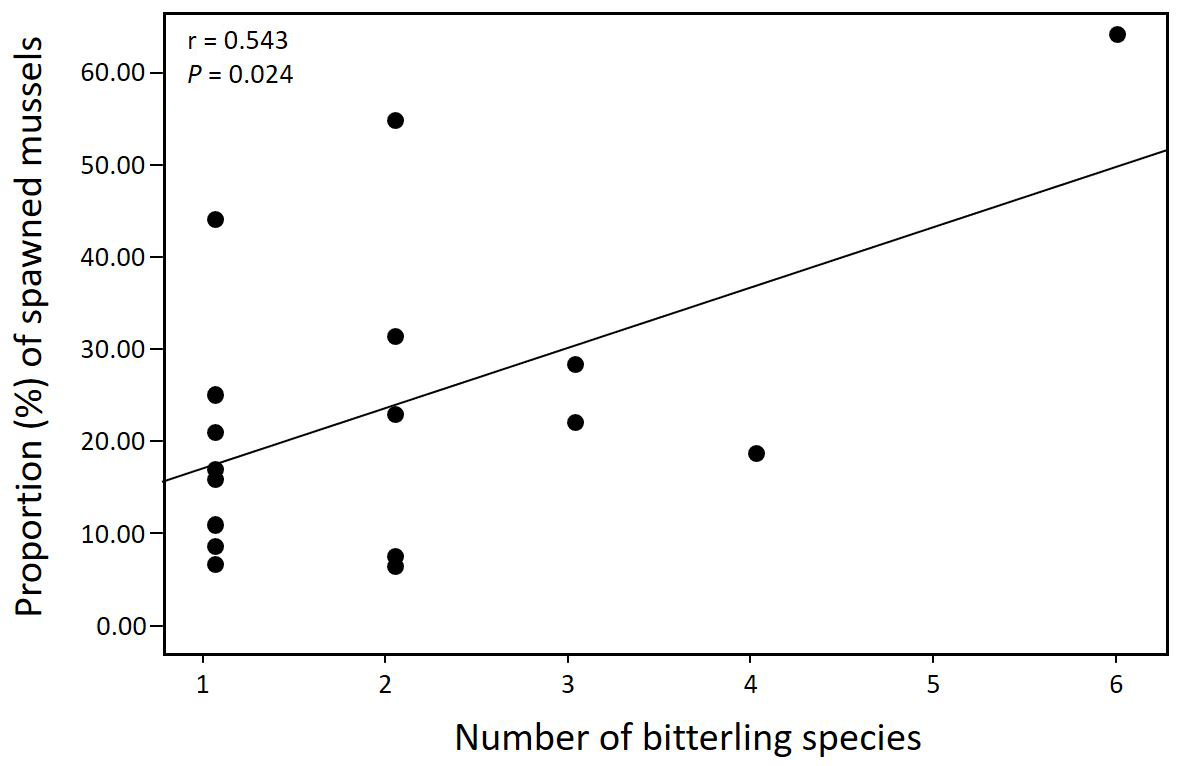

Supplement: Supplementary file 3 — Figure S3. [file ECE3-14-e11142-s004.tif]
