# Supplementary material for: Host size matters for reproduction: Evolution of spawning preference and female reproductive phenotypes in mussel‐symbiotic freshwater bitterling fishes
Source: Ecol Evol. 2024 Mar 11;14(3):e11142. doi: 10.1002/ece3.11142 (PMC10927361; doi:10.1002/ece3.11142)
Supplement: Supplementary file 4 — Table S1. [file ECE3-14-e11142-s002.docx]

**Figure captions**

**Figure S1** Results of restriction fragment length polymorphism (RFLP) experiments for the eight Korean bittering fish species. (a) left: DNA bands produced from mtDNA cyt *b* PCR reaction (lanes 2-9) in gel electrophoresis, right: DNA bands obtained from RFLP experiments using *Hin*P1l (G'CGC) enzyme (lanes 2-9) in gel electrophoresis, (b) left: DNA bands produced from cyt *b* PCR reaction (lanes 2-6) in gel electrophoresis, right: DNA bands obtained from RFLP experiments using *Rsa*l (GT'AC) enzyme (lanes 2-6) in gel electrophoresis.

**Figure S2** Relationship between mussel density and proportion of spawned mussels (Pearson correlation; *r* = 0.307, *P* = 0.265).

**Figure S3** Relationship between number of bitterling fish species and proportion of spawned mussels (Pearson correlation; *r* = 0.543, *P* = 0.024).

**Appendix Table S1.** Information of sampling localities, river basins, location codes and latitude/longitude in this study.

| **River basin** | **Location code** | **Location** | **Latitude** | **Longitude** |
| --- | --- | --- | --- | --- |
| Imjin-Hantan River basin | YI | Yeoncheon Imjin-gang | 38°02'26"N | 127°01'31"E |
|  | CH | Cheorwon Hantan-gang | 38°12'42"N | 127°15'56"E |
| North Han River basin | II | Inje Inbuk-cheon | 38°11'54"N | 128°12'27"E |
|  | GG | Gapyeong Gapyeong-cheon | 37°53'03"N | 127°32'40"E |
|  | HN | Hongcheon Naechon-cheon | 37°48'07"N | 128°05'19"E |
|  | HD | Hongcheon Deokchi-cheon | 37°41'52"N | 127°55'42"E |
|  | HM | Hongcheon Myeongseong-cheon | 37°38'33"N | 127°37'29"E |
| South Han River basin | HG | Hoengseong Geumgye-cheon | 37°32'38"N | 127°58'15"E |
|  | HI | Hoengseong Iri-cheon | 37°25'45"N | 127°51'28"E |
|  | GS | Ganhyeon Samsan-cheon | 37°21'54"N | 127°49'02"E |
|  | JG | Jeongseon Golji-cheon | 37°28'24"N | 128°43'46"E |
|  | JJ | Jeongseon Joyang-gang | 37°22'05"N | 128°37'11"E |
|  | PP | Pyeongchang Pyeongchang-gang | 37°16'57"N | 128°20'47"E |
|  | YO | Yeongwol Okdong-cheon | 37°07'36"N | 128°34'48"E |
|  | DY | Danyang Yeongok-cheon | 37°03'24"N | 128°23'19"E |
|  | DM | Danyang Maepo-cheon | 37°03'15"N | 128°18'03"E |
|  | GD | Goesan Dal-cheon | 36°40'57"N | 127°46'30"E |
