# Supplementary material for: Host size matters for reproduction: Evolution of spawning preference and female reproductive phenotypes in mussel‐symbiotic freshwater bitterling fishes
Source: Ecol Evol. 2024 Mar 11;14(3):e11142. doi: 10.1002/ece3.11142 (PMC10927361; doi:10.1002/ece3.11142)
Supplement: Supplementary file 5 — Table S2. [file ECE3-14-e11142-s003.docx]

**Appendix Table S2.** Results of Dunn’s multiple comparison tests after nonparametric Kruskal-Wallis tests for mean number of eggs/larvae per mussel (above diagonal) and mean shell length of spawned mussels (below diagonal).

| **Dunn’s multiple comparison tests (post hoc)** | | | | | | | **Kruskal-Wallis tests** | | | |
| --- | --- | --- | --- | --- | --- | --- | --- | --- | --- | --- |
| Species | *A. rhombeus* | *A. yamatsute* | *R. pseudosericeus* | *R. uyekii* | *T. lanceolatus* | *T. signifer* | Variable | *H* | *df* | *p*-value |
| *A. rhombeus* | - | ******* | ****** | ns | ****** | ******* | Mean number of offspring per mussel | 124.67 | 5 | < 0.001 |
| *A. yamatsute* | ns | - | ns | ******* | ******* | ns |  |  |  |  |
| *R. pseudosericeus* | ******* | ******* | - | ******* | ******* | ns |  |  |  |  |
| *R. uyekii* | ns | ns | ******* | - | ns | ******* | Mean shell length of spawned mussels | 114.61 | 5 | < 0.001 |
| *T. lanceolatus* | ****** | ns | ******* | ns | - | ******* |  |  |  |  |
| *T. signifer* | ******* | ns | ******* | ns | ns | - |  |  |  |  |

Asterisks indicate significant differences (**: *p* <0.005, ***: *p* < 0.001) after the Bonferroni correction; ns: not significant; *H*: *H* test value; *df*: degree of freedom.
